# The Influence of Cimicifuga Racemosa on Parturition

**Published:** 1885-04

**Authors:** J. Suydam Knox

**Affiliations:** Adjunct Professor of Obstetrics and Diseases of Children, Rush Medical College


### ARTICLE II.

### THE INFLUENCE OF CIMICIFUGA RACEMOSA ON PARTURITION.

*By* J. SUYDAM KNOX, A. M., M. D., *Adjunct Professor of Obstetrics and Diseases of Children, Rush Medical College.*

[Read before the Chicago Gynecological Society, March 20, 1885.]

Cimicifuga racemosa is no new drug. Ever since the paper of Dr. Young, in 1831 (*Amer. Jour. Med. Sci.*, vol. 9), black cohosh has been largely used by American practitioners.

On reviewing the literature of the subject, quite uniform testimony is found from eclectic, homœopathic and regular physicians, as to the sedative and anti-spasmodic action of the drug. This testimony is strongest among eclectic physicians, but is sufficiently voluminous among regular prescribers to command attention.

This clinical proving asserts the positive value of cimicifuga in chorea, spasmodic cough, congestive amenorrhœa and dysmenorrhœa, and in rheumatism. Eclectic physicians in addition claim for it oxytocic properties, recommending it as a substitute for ergot (King & Lloyd's Amer. Dispens., 15th Edit.)

It is surprising that the physiological action of a drug so long and widely used has not yet been ascertained, and its special therapeutic value determined.

About three years ago I first commenced to use cimicifuga racemosa in obstetric practice, my attention having been called to it by an obstetrician of large experience of this city. He stated positively that the use of black cohosh, on account of its favorable influence on labor, had shortened his average attendance in the lying-in room at least two hours.

I at first prescribed the drug to all cases that I was called upon to attend, but later, on account of certain precipitate labors, only to primiparæ, and those multiparæ, who had a previous history of dystocia.

My cases number 150: 57 primiparæ and 93 multiparæ. Considering labor as commencing as soon as pains are regular, its average duration among primiparæ was six and one-fourth hours, and among multiparæ exactly three hours.

Considering the second stage of labor as beginning with full dilatation of the external os, its average duration among the primiparæ was one hour and forty-five minutes, and among the multiparæ only twenty-seven minutes.

In reviewing my notes of the above 150 cases, I have come to the following conclusions:

1. Cimicifuga has a positive sedative effect upon the parturient woman, quieting reflex irritability. Nausea, pruritus and insomnia, so common in the last six weeks of pregnancy, are always bettered, and often disappear, under its administration.

2. Cimicifuga has a positive anti-spasmodic effect upon the parturient woman. The neuralgic cramps and irregular pains of the first stage of labor are ameliorated, and often altogether abolished. In fact, during the first indiscriminate use of the drug in all cases, I had the mortification, with a few women, of terminating the labor so precipitately, and without prodromic symptoms, as to be unable to reach the bedside before the birth.

3. Cimicifuga relaxes uterine muscular fibre and the soft parts of the parturient canal, by controlling muscular irritability, thus facilitating labor and diminishing risks of laceration.

4. Cimicifuga increases the energy and rhythm of the pains in the second stage of labor.

5. It is my belief that cimicifuga, like ergot, maintains a better contraction of the uterus after delivery. It is my habit, however, to administer 15 to 30 minims of fld. ext. ergot after the birth of the fœtal head, and I have had but few opportunities of testing this effect of the cohosh.

To illustrate the above propositions, the following cases are cited:

*Case 1.*—Mrs. S. Second labour. First labour protracted through two days. Finally puerperal eclampsia and a forceps delivery. November 17, 1884, consulted me for uterine neuralgia, with insomnia and pruritus. Prescribed fld. ext. cimicifuga, 15 minims night and morning. Urine, normal. Complete relief of bad symptoms followed. Normal labour occurred six weeks thereafter. Second stage, forty-five minutes.

*Case 2.*—Mrs. B. Second labour occurred December 23, 1884. Eighteen months before I attended her in her first labour, which lasted nine hours, and finally used forceps. She had refused to take cohosh. She cheerfully took the drug the last four weeks of her second pregnancy. Pains came on without prodromic symptoms, and the babe was born before my arrival, fifteen minutes after the first indications of labour.

*Case 3.*—Mrs. C. Fifth labour, November 20, 1884. As all previous labours had been instrumental, I gave cohosh night and morning for six weeks before confinement. She evidently got too much of the drug, for a week before delivery I was sent for on account of bearing down without pains, and found the os dilated fully three inches. This condition existed seven days, when the waters broke for want of support, and labour came on and was terminated in ninety minutes.

*Case 4.*—Mrs. K. Third labour, February 11, 1884. Two previous labours tedious and instrumental. Took cohosh each night for four weeks before present labour. First stage, three hours; second stage, thirty minutes; delivery normal.

*Case 5.*—Mrs. L. Third labour, January, 1885. First labour, ninety hours; second labour, forty-five hours, and delivery instrumental. Took cohosh, on account of an error in calculation, each night for two months before third labour. Like case 3, she took too much of the drug, and three days before labour I was called to see her on account of bearing down without pains. Found the os widely dilated and membranes protruding. Labour came on suddenly the third day thereafter, and delivery occurred within an hour after the pains set in.

*Case 6.*—Mrs. R. Second labour, February 22, 1884. First labour, three years before, was extremely tedious in its first stage, lasting forty hours, with extreme pain before the os dilated. Second labour, under the influence of cohosh, came on without prodromes, and was accomplished within an hour.

*Case 7.*—Mrs. D. First labour. Under a dread of pain, she took twice the quantity I ordered, for two weeks before delivery. Labour came on precipitately, and the child was born in forty-five minutes.

I might enumerate a score of cases, were it not a tedious repetition.

My method of administration has been to give 15 mimins of the fld. ex. of cimicifuga in co. syr. of sarsaparilla each night for four weeks before the expected confinement.

One fluid ounce of the fluid ext. cimicifuga to three fluid ounces co. syr. sarsaparilla; dose, one teaspoonful, makes just the required quantity.

